# Examining Unlock Journaling with Diaries and Reminders for In Situ Self-Report in Health and Wellness

**DOI:** 10.1145/2858036.2858360

**Published:** 2016-05-07

**Authors:** Xiaoyi Zhang, Laura R. Pina, James Fogarty

**Affiliations:** 1 Computer Science & Engineering, DUB Group, University of Washington; 2 Human Centered Design & Engineering, DUB Group, University of Washington

**Keywords:** Personal informatics, self-tracking, experience sampling

## Abstract

In situ self-report is widely used in human-computer interaction, ubiquitous computing, and for assessment and intervention in health and wellness. Unfortunately, it remains limited by high burdens. We examine *unlock journaling* as an alternative. Specifically, we build upon recent work to introduce single-slide unlock journaling gestures appropriate for health and wellness measures. We then present the first field study comparing unlock journaling with traditional diaries and notification-based reminders in self-report of health and wellness measures. We find unlock journaling is less *intrusive* than reminders, dramatically improves *frequency* of journaling, and can provide equal or better *timeliness*. Where appropriate to broader design needs, unlock journaling is thus an overall promising method for in situ self-report.

## Introduction and Motivation

The use of mobile devices for prompted in situ self-report has become common across a variety of disciplines, sometimes called experience sampling [3,15,23], diaries [8,34,36,41], or ecological momentary assessment [21,40,44]. Applications of these methods are too broad to enumerate, but research in the CHI community has included studies of mobile device adoption [34], of time management and interruptions [17,24,26,35,38], of relating sensed behavior traces to explicit reports [20,27], of location privacy preferences [13], and informing and evaluating mobile and ubiquitous computing applications [7,11,12,14,41]. The CHI community is also actively exploring self-tracking through the lenses of personal informatics [32], the Quantified Self [10], and lived informatics [16,18,37]. We are particularly interested in the increasing applications of these techniques to self-monitoring in health and wellness (e.g., [6,28]).

Unfortunately, potential benefits of these techniques remain limited by their high burdens. Quantified Selfers report the burdens of self-tracking often create a fatigue that leads them to abandon self-tracking [10]. Experience sampling research has found the frequency of prompts must be limited to mitigate annoyance they create [19,27]. Intrusiveness of prompts can also be reduced using context awareness and decision theory methods to focus prompts at appropriate times and reduce the total number of prompts [25,26,38]. Health and wellness applications face the additional challenge that the act of journaling is often itself intended to promote mindfulness and self-monitoring (i.e., self-report serves both assessment and treatment [29]). Too much of a focus on sensing, automating data collection, or minimizing journaling may therefore undermine the entire purpose [42].

**Figure 1:**
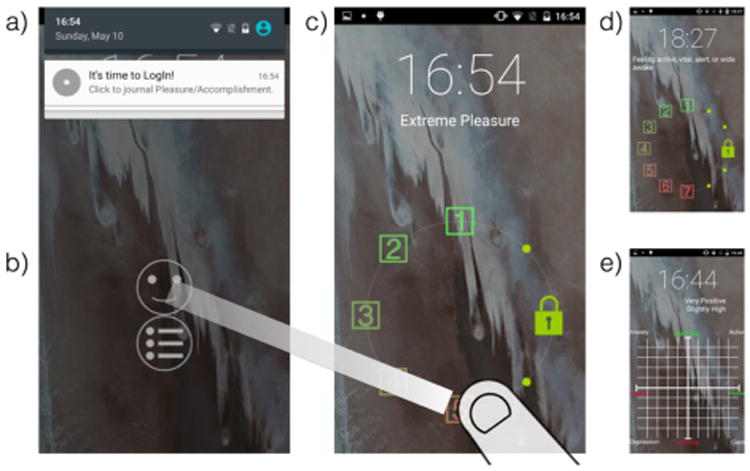
We examine unlock journaling, in which unlocking a phone also journals an in situ self-report. We present the first study comparing to diaries and notification-reminders.

Initial methods used beepers (i.e., pagers) to prompt people to complete paper diaries [15]. This same basic approach continues in mobile interfaces that present a survey with multiple questions requiring minutes to complete. In contrast, recent work explores more lightweight journaling interfaces. Choe et al.'s SleepTight uses a phone lockscreen widget for journaling of potential sleep disruptors with as little as a single tap [9]. We build upon work by Truong et al., repurposing the otherwise wasted action of unlocking a phone (i.e., the slide to unlock gesture) to allow a small optional action [46]. Figure 1 illustrates our LogIn system, which modifies the unlock gesture to enable single-slide unlock journaling of health and wellness self-reports for sleepiness [22], pleasure and accomplishment [31], or mood [39].

Although repurposing the unlock gesture is appealing in its potential to reduce burdens of journaling, no prior work has compared this with traditional approaches. Researchers and designers thus lack information regarding how unlock journaling for health and wellness might be expected to perform in practice. We therefore present a field study in which 24 people each journal a health and wellness measure for 18 days. We compare unlock journaling with diaries and notification-based reminders, finding that unlock journaling is less *intrusive* than reminders, dramatically improves *frequency* of journaling, and can provide equal or better *timeliness*. Where appropriate to broader design needs, unlock journaling is thus a promising method for in situ self-report.

The specific contributions of this work therefore include:

- We expand the design of single-slide unlock journaling gestures to support (1) selection among multiple measures to journal, and (2) journaling a two-dimensional measure.
- We present the first field study comparing unlock journaling with traditional diaries and notification-based reminders for self-report of health and wellness measures.

## Related Work

The CHI community adopted diary studies as an alternative to field observation, which requires a researcher be present [36]. People often forget to journal, so methods evolved to provide reminders via beepers (i.e., pagers) [15], mobile devices [3], and text messages [1]. As devices became more common and capable, techniques have been updated to leverage advances in mobile media capture [8,34], automatic sensing [19,25], mobile notifications [5], and context awareness [25,26,38]. Enabled by these advances, an important thread of research has examined how those advances impact in-situ self-report [3,5,8,43].We similarly contribute by examining the potential of unlock journaling as a new low-burden approach to in situ self-report, focusing on health and wellness applications.

We build directly on Truong et al.'s Slide to X technique [46]. This technique assigns a dual purpose to the unlock gesture, aiming to preserve its extreme simplicity while having that same gesture perform a simple, optional task. They propose Slide to QuantifySelf, but do not field such an application, nor do they compare to data collection with traditional diaries or notification-based reminders. We extend the single-slide gestures illustrated by Truong et al. to support common health and wellness measures. We also present the first field study comparing single-slide unlock journaling to traditional approaches. Additional examples of lightweight lockscreen interaction include small crowdsourcing tasks in Twitch [47], a crossing-based interface for email review in ProactiveTasks [2], and single-tap journaling via a lockscreen widget in SleepTight [9]. None of these provide the guidance we contribute on how single-slide unlock journaling might perform for a variety of health and wellness measures.

## Design of Login

**Figure 2:**
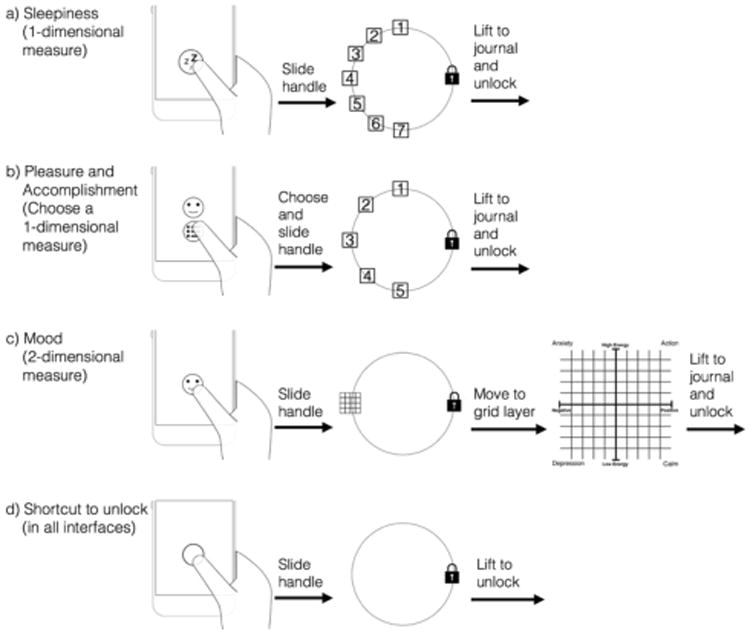
We design single-slide unlock gestures to support journaling of sleepiness (i.e., a rating scale), pleasure and accomplishment (i.e., choosing the scale to journal), and mood (i.e., a two-dimensional scale). All designs make journaling optional, preserving the simplicity of the default unlock.

LogIn is our implementation of unlock journaling, focused on validated health and wellness measures selected to be representative of needs in this space [22,31,39]. This section details our design of singe-slide unlock journaling in LogIn. Our first measure is the Stanford Sleepiness Scale, a 7-point one-dimensional scale administered multiple times per day to identify sleep debt and patterns of alertness [22]. Such a scale is supported by the original Likert-type design in Slide to X [46], though we modify the design to preserve Android conventions and avoid thumb occlusion. Drop targets appear when a person touches the unlock handle, a text explanation of a target is provided by sliding the handle to it, and releasing the handle then simultaneously journals the selection and unlocks the phone (Figure 1d, 2a).

Our second measure is for pleasure and accomplishment, implemented as a pair of 5-point one-dimensional scales. A person working with a therapist to assess and treat depressive symptoms may journal whether activities create a sense of pleasure or accomplishment, as such activities are important to behavioral activation in managing symptoms [4,31,33]. The original Slide to X interactions do not support single-slide selection of what to journal, so we introduce choosing from *multiple handles* to slide. A person chooses which scale to journal by touching the appropriate handle, then the interaction proceeds as before (Figure 1b, 1c, 2b).

Our third measure is Russell's Affect Grid, a two-dimensional measure of mood (i.e., pleasure and arousal) appropriate for journaling throughout the day [39]. The original Slide to X interactions do not support single-slide gesturing to journal in a two-dimensional scale. This is challenging in part because of the need to allow selection from the grid while preserving the default slide to unlock interaction (i.e., Figure 2d). We resolve this by introducing *layers* in the slide interaction. A person slides the handle to a grid icon, which activates the grid layer, then slides to the desired value and releases (Figure 1e, 2c), simultaneously journaling and unlocking.

Journaling is always optional, and all designs preserve the default unlock gesture (Figure 2d). Journaling is therefore visible and easy but not required, as required journaling could result in people recording less reliable data while attempting to quickly access their phone. Although it is important our field evaluation is based in commonly-used measures, we also again note these measures were selected to be representative of needs in this space. Other health and wellness measures could likely be supported through these interactions or through designs based on their insights (e.g., choosing among layers via multiple slide targets).

### Implementation

LogIn is implemented for Android, which provides a public API for customizing the lockscreen. Unlock journaling thus replaces the default unlock gesture (i.e., is not an additional step in the interaction). Any system password is preserved and entered after the unlock gesture (i.e., at the same point in the overall interaction sequence).

## Field Evaluation

Our evaluation examines unlock journaling in a field study focused on health and wellness self-report, comparing to traditional diaries and notification-based reminders.

### Design and Procedure

We use a 2×3 within-subjects design, examining the impact of interfaces with *unlock journaling* (with, without) and *notifications* (none, traditional, aggressive) on *intrusiveness*, *frequency*, and *timeliness* of journaling in health and wellness.

*Traditional notifications* conditions generated a platform notification reminding the participant to journal (Figure 1a). *Aggressive notifications* added sound/vibration. Notifications were generated every 30 minutes in a configurable 12-hour window, applying a small random jitter to avoid grouping with any notifications from other applications at the same approximate time. The participant configured this window at the beginning of the study. For comparison, unlock journaling was only presented during this same window.

#### Base Functionality

All conditions provided an app which allowed journaling at will (i.e., participants could journal at any time, without needing to wait for a notification or first lock their phone). A simple app also visualized a participant's data, allowing them to view their journal at will. Note that the condition *without unlock journaling* and *none notifications* is a traditional *diary* (i.e., can enter and view data, but does not otherwise surface itself or remind participants to journal).

#### Journaling Domain

To maximize participant interest in the task, we screened participants for interest and allowed them to choose their journaling domain from sleepiness, pleasure and accomplishment, or mood. Each participant used different interfaces for journaling in their chosen domain over the course of their participation.

#### Procedure

Participants used their own Android phones to journal with LogIn over the course of 18 days. Each of the 6 conditions was used for 2 consecutive days, with a rest day between conditions. The LogIn app automatically switched interface conditions at midnight. To account for possible carryover effects (e.g., fatigue, learning, varying interest), we counterbalanced conditions using a balanced Latin Square.

Participation began with a brief interview, during which we installed LogIn on the participant's phone. We explained the study, then they chose their domain and configured their 12-hour journaling window. We emphasized compensation was not dependent on frequency of interaction, only a need to leave LogIn installed and return for a brief exit interview. Participants were compensated with a $20 gift card.

### Participants

We recruited through local email lists, linking to a questionnaire screening for use of a phone running Android 4.2 or above and for interest in three domains. From the eligible pool, we randomly selected 24 participants, reporting an average age of 26.1 years (min 19, max 47, med 24), 14 male, 10 female. 11 chose to journal sleepiness, 6 pleasure and accomplishment, and 7 mood. Reporting motivation to journal in their chosen domain on a 7-point Likert scale, 15 reported motivation 7, 7 motivation 6, and 2 motivation 5.

### Results

Participants journaled an average of 147 entries in 12 days (min 36, max 255, med 149), or 12.2 per day. When unlock journaling was active in the interface condition, they unlocked without journaling an average of 228 times, or 38 per day. We analyze their reported *intrusiveness* of conditions and their *frequency* and *timeliness* of journaling by condition.

**Figure 3:**
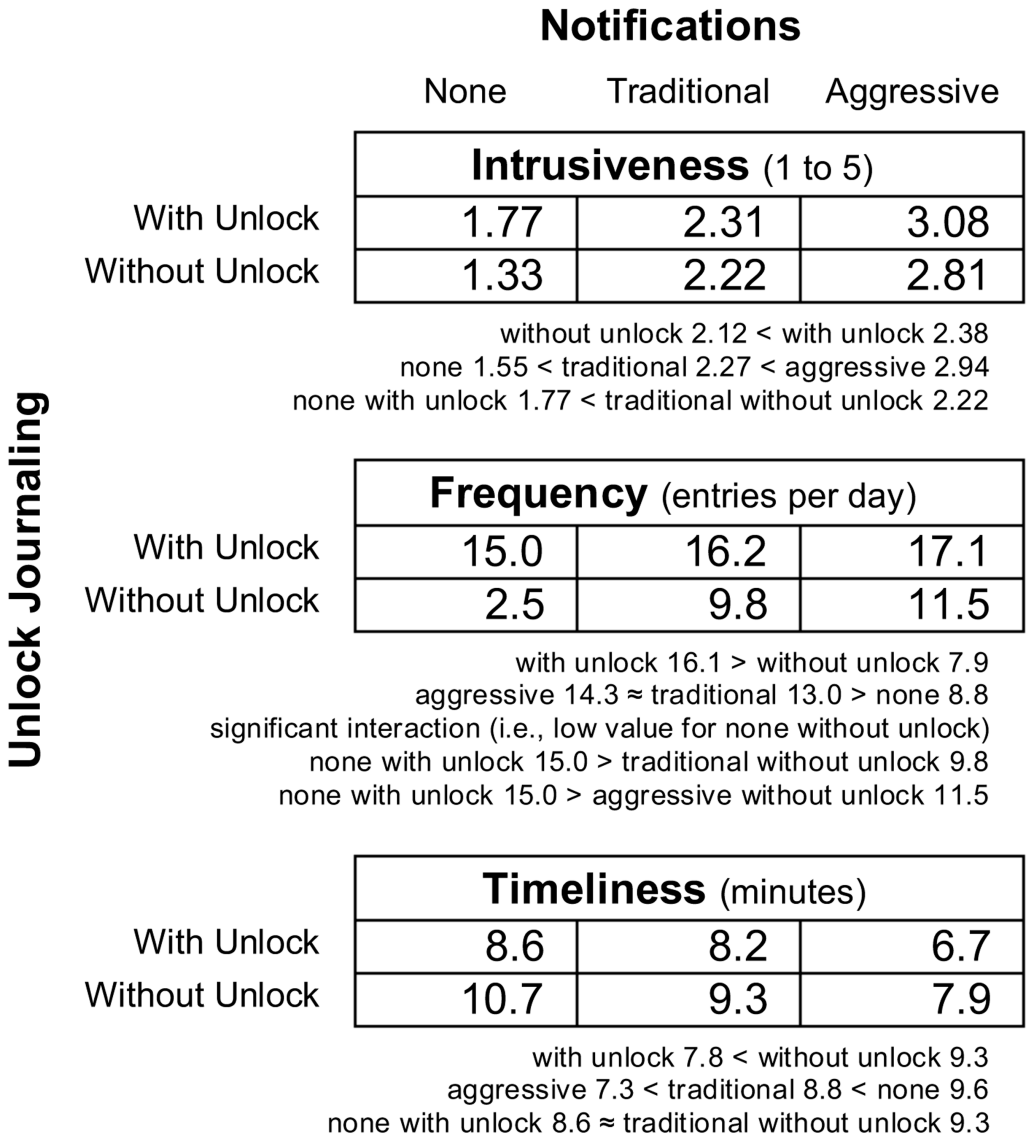
Summary of analyses, all as least squares means. Compared to notifications, unlock journaling is less *intrusive*, dramatically *improves frequency*, with equal or better *timeliness*.

We use mixed-model analyses of variance, with *participant* as a random effect (i.e., accounting for individual differences). We model *domain* and *study day*, but find no significant results (i.e., no differences by journaling domain and no carryover effects). We model *unlock journaling*, *notifications*, and their interaction. Figure 3 presents our primary results. To control for other independent variables, means reported in Figure 3 and this section are all least squares means.

#### Intrusiveness

At the end of each 12-hour study window, an alert asked participants to rate *intrusiveness* of that day's condition on a 5-point Likert scale. We find the addition of *unlock journaling* is reported as more *intrusive* (2.12 vs. 2.38, *F*_1,258_ = 11.7, *p* ≈ .001). We find a main effect for *notifications* (*F*_2,258_ = 12.7, *p* < .001), and Tukey's HSD finds *aggressive* (2.94) is reported as more *intrusive* than *traditional* (2.27, *F*_1,258_ = 51.3, *p* < .001), with both more *intrusive* than *none* (1.55, *F*_1,258_ = 218, *p* < .001, *F*_1,258_ = 57.9, *p* < .001).

Of particular interest is a contrast between *unlock journaling* and *notifications* (e.g., a designer choosing one or the other to enhance a *diary* that has neither). Tukey's HSD finds addition of *notifications* is more intrusive than *unlock journaling* (2.22 vs. 1.77, *F*_2,258_ = 11.8, *p* ≈ .001). Part of this may be that unlock journaling is surfaced during normal interaction, which P18 explained as: “*I have so many different reminder and buzzing things and don't know which one is the journal reminder. But if I see this lockscreen, I know I am supposed to do it … it is very easy.*”

#### Frequency

We define *frequency* as the number of entries a person journals in a day. We find the addition of *unlock journaling* doubles *frequency* (16.1 vs. 7.9 entries per day, *F*_1,258_ = 188, *p* < .001). We find a main effect for *notifications* (*F*_2,258_ = 31.4, *p* < .001). Tukey's HSD finds *aggressive* (14.3) and *traditional* (13.0) yield greater *frequency* than *none* (8.8, *F*_1,258_ = 57.5, *p* < .001, *F*_1,258_ = 33.6, *p* < .001), but not significantly different from each other (*F*_1,258_ = 3.19, *p* ≈ .075). Part of *unlock journaling*'s higher *frequency* and a lack of improvement with *aggressive notifications* may be that notifications become less effective if delivered too often or too aggressively, contributing to *intrusiveness* instead of increasing *frequency*, as P7 explained: “*for the days there were many notifications, I feel like ‘Ugh, leave me alone’*”.

We find a significant interaction between *unlock journaling* and *notifications* (*F*_2,258_ = 13.1, *p* < .001). Tukey's HSD finds the *diary* condition (i.e., *none without unlock*) yields significantly less *frequency* than other conditions (2.5, all pairs *p* < .001). The *diary* condition provided no reminder to journal, and some reported forgetting entirely (e.g., P9 said “*I rarely remember if I don't have any notifications and (the lockscreen) is shown nothing. You can see from what I did … I probably hardly journal anything.*” Of 13 days that participants had a *frequency* of 0, 10 are in the *diary* condition (including 1 from p1, who did not journal on 3 days and made only 36 entries over the entire study).

The contrast between *unlock journaling* and *notifications* is again of particular interest, and we find that *unlock journaling* improves *frequency* significantly more than *traditional notifications* (15.0 vs. 9.8, *F*_1,258_ = 25.6, *p* < .001) or *aggressive notifications* (15.0 vs. 11.5, *F*_1,258_ = 11.5, *p* ≈ .001).

#### Timeliness

Our final analysis examines *timeliness* separately from *frequency*, as some domains may require journaling near a particular time. Considering only cases where a self-report was provided, we examine average proximity to the nearest 30-minute interval (i.e., when notification was or would have been made). We define proximity as two-sided, ranging from 0 to 15. We exclude 13 days with *frequency* 0.

We find the addition of *unlock journaling* improves *timeliness* (7.8 vs. 9.3, *F*_1,245_ = 49.5, *p* < .001). We find a main effect for *notifications* (*F*_2,245_ = 42.9, *p* < .001), and Tukey's HSD finds *aggressive* (7.3) improves *timeliness* over *traditional* (8.8, *F*_1,245_ = 33.8, *p* < .001), and both improve over *none* (9.6, *F*_1,245_ = 83.1, *p* < .001, *F*_1,245_ = 11.9, *p* < .001).

We are again particularly interested in a contrast between *unlock journaling* and *notifications* (e.g., a designer choosing one or the other to enhance a *diary* that has neither). Tukey's HSD finds addition of *unlock journaling* or *notifications* to a *diary* to be indistinguishable in their impact on *timeliness* (8.6 vs. 9.3, *F*_2,245_ = 4.28, *p* ≈ .040, but failing the test's adjustment for multiple comparisons).

## Discussion

If choosing between unlock journaling or notifications, our results suggest unlock journaling will be perceived as less *intrusive* (1.77 vs. 2.22), yield greater *frequency* of journaling (15.0 vs. 9.8), with comparable *timeliness* (8.6 vs. 9.3). Unlock journaling can also be combined with notifications, improving *frequency* and *timeliness* with a small increase in *intrusiveness.* These results motivate additional longer-term comparisons and studies of other characteristics of unlock journaling (e.g., different reporting windows, long-term fatigue, reliability of self-report collected in this manner).

Unlock journaling is limited to self-report that can be reduced to single-swipe gestures. We have extended the space of such gestures, but interesting opportunities for future work include pairing unlock journaling with other methods (e.g., with wearable eating detection [45] for lightweight verification of sensed data). LogIn also adds some complexity to a phone's unlocking mechanism. Although not a concern for our participants, this complexity could be a challenge for some.

Finally, Lathia et al. note the design of experience sampling implies bias in the resulting data [30]. This is unavoidable, as every method has a bias, but should be considered in applying a method. Experience sampling is already biased toward times a person can respond, and unlock journaling may magnify this to times when they are already accessing their phone. Depending on the application, this is potentially offset by the greater frequency of journaling.

There remain significant opportunities to extend this first field study comparing unlock journaling with traditional diaries and notification-based reminders in self-report of health and wellness measures. But our overall results suggest that, where appropriate to broader design needs, unlock journaling is a promising method for in situ self-report.
